# Perinatal and Postpartum Health Among People With Intellectual and Developmental Disabilities

**DOI:** 10.1001/jamanetworkopen.2024.28067

**Published:** 2024-08-15

**Authors:** Lindsay Shea, Molly Sadowsky, Sha Tao, Jessica Rast, Diana Schendel, Arina Chesnokova, Irene Headen

**Affiliations:** 1 A.J. Drexel Autism Institute, Drexel University, Philadelphia, Pennsylvania; 2 Division of Academic Specialists, University of Pennsylvania, Philadelphia; 3 Dornsife School of Public Health, Drexel University, Philadelphia, Pennsylvania

## Abstract

**Importance:**

Small, geographically limited studies report that people with intellectual and developmental disabilities (IDD) have increased risk for serious pregnancy-related and birth-related challenges, including preeclampsia, preterm birth, and increased anxiety and depression, than their peers. United States–based population-level data among people with IDD are lacking.

**Objectives:**

To identify perinatal and postpartum outcomes among a national, longitudinal sample of people with IDD enrolled in public health insurance, compare subgroups of people with IDD, and compare outcomes among people with IDD with those of peers without IDD.

**Design, Setting, and Participants:**

This retrospective cohort study used national Medicaid claims from January 1, 2008, to December 31, 2019, for 55 440 birthing people with IDD and a random sample of 438 557 birthing people without IDD. Medicaid funds almost half of all births and is the largest behavioral health insurer in the US, covering a robust array of services for people with IDD. Statistical analysis was performed from July 2023 to June 2024.

**Exposure:**

People who had a documented birth in Medicaid during the study years.

**Main Outcome and Measures:**

Perinatal outcomes were compared across groups using univariate and multivariate logistic regression. The probability of postpartum anxiety and depression was estimated using Kaplan-Meier and Cox proportional hazards regression.

**Results:**

The study sample included 55 440 birthing people with IDD (including 41 854 with intellectual disabilities [ID] and 13 586 with autism; mean [SD] age at first delivery, 24.9 [6.7] years) and a random sample of 438 557 birthing people without IDD (mean [SD] age at first delivery, 26.4 [6.3] years). People with IDD were younger at first observed delivery, had a lower prevalence of live births (66.6% vs 76.7%), and higher rates of obstetric conditions (gestational diabetes, 10.3% vs 9.9%; gestational hypertension, 8.7% vs 6.1%; preeclampsia, 6.1% vs 4.4%) and co-occurring physical conditions (heart failure, 1.4% vs 0.4%; hyperlipidemia, 5.3% vs 1.7%; ischemic heart disease, 1.5% vs 0.4%; obesity, 16.3% vs 7.4%) and mental health conditions (anxiety disorders, 27.9% vs 6.5%; depressive disorders, 32.1% vs 7.5%; posttraumatic stress disorder, 9.5% vs 1.2%) than people without IDD. The probability of postpartum anxiety (adjusted hazard ratio [AHR], 3.2 [95% CI, 2.9-3.4]) and postpartum depression (AHR, 2.4 [95% CI, 2.3-2.6]) was significantly higher among autistic people compared with people with ID only and people without IDD.

**Conclusions and Relevance:**

In this retrospective cohort study, people with IDD had a younger mean age at first delivery, had lower prevalence of live births, and had poor obstetric, mental health, and medical outcomes compared with people without IDD, pointing toward a need for clinician training and timely delivery of maternal health care. Results highlight needed reproductive health education, increasing clinician knowledge, and expanding Medicaid to ensure access to care for people with IDD.

## Key Points

**Question:**

How do perinatal and postpartum outcomes differ for birthing people with intellectual and developmental disabilities (IDD) compared with their peers without IDD?

**Findings:**

This cohort study including data from 55 440 birthing people with IDD enrolled in Medicaid found that birthing people with IDD had a younger mean age at first delivery (24.9 years vs 26.4 years) compared with people without IDD. In addition, birthing people with IDD had higher risks for multiple medical and mental health conditions (gestational diabetes, 10.3% vs 9.9%; gestational hypertension, 8.7% vs 6.1%; preeclampsia, 6.1% vs 4.4%).

**Meaning:**

This study suggests that clinician training and resources for birthing people with IDD and their families or caregivers are needed to support reproductive health care and improve outcomes for birthing people, which supports outcomes for their children and families as well.

## Teaser

This cohort study examines perinatal and postpartum outcomes among a national, longitudinal sample of people with intellectual and developmental disabilities enrolled in public health insurance and compares outcomes with those of peers without intellectual and developmental disabilities.

## Introduction

Intellectual and developmental disabilities (IDD) are characterized by neurodevelopmental differences in intellectual functioning and adaptive behavior and challenges with social interaction and communication that may affect an individual throughout the life course. Identifying opportunities to improve health outcomes among the 7 million people with IDD, including autism and intellectual disabilities (ID), living in the US^1^ is a key component of improving quality of care and health care system effectiveness and efficiency. Although research suggests that the number of people with IDD who give birth is increasing,^2^ research about health outcomes across pregnancy, childbirth, and postpartum periods for this population is limited. Research into populations with IDD has historically focused on children and disproportionately on male individuals, given the prevalence information (mostly generated during childhood), but there is growing attention on outcomes among people with IDD as they age into adulthood, especially during periods of higher risk for adverse health outcomes, such as the perinatal period.^3^

Emerging research predominantly from Scandinavian and European countries on pregnancy, childbirth, and postpartum periods among birthing people with IDD highlights critical disparities. For example, birthing people with IDD tend to be younger compared with their peers without IDD^4^ and have a higher likelihood of being single parents,^5^ which are both risk factors for isolated parenting experiences and victimization that heighten risk for adverse mental health outcomes and other health outcomes. Studies have also found higher rates of prenatal and postpartum depression among people with IDD compared with people without IDD.^6,7,8,9^ The experiences of pregnancy and the postpartum period can exacerbate preexisting mental health issues, and reports have indicated higher rates of psychiatric conditions among birthing people with IDD.^10,11^ Higher rates of preterm birth among people with IDD escalates the risk of maternal mental health issues, such as depression and posttraumatic stress disorder (PTSD), affecting birthing people and their infants in the short and long term.^12^ Research using national US samples to examine these issues is needed to complement qualitative and smaller sample investigations^13,14^ and to inform opportunities for improving policies and programs.

Characteristics associated with IDD, including social and communication differences and sensory sensitivities, may increase vulnerability to the stressors and changes experienced physically and mentally throughout the perinatal period, emphasizing the need for services and supports to address increased risk.^14,15^ Subgroups of individuals with IDD (ie, autistic people) have reported sensory overload, a lack of control, and challenges in effectively engaging with medical care professionals, including obstetrician-gynecologists.^6,15,16,17,18,19^ Lack of effective support for birthing people with IDD can lead to increased risk for custody loss, particularly among people living in poverty who face barriers to accessing adequate care.^20^

Examination of perinatal health in the US, specifically the Black maternal health crisis, prioritizes research that includes diverse racial and ethnic groups. Black birthing people in the US experience disproportionately higher rates of pregnancy-related death, maternal morbidity, and adverse birth outcomes compared with White birthing people, even among Black birthing people accessing the same system of care as their White peers.^21,22^ Among women of racial and ethnic minority groups with IDD, increased risk for mental health comorbidities, pregnancy complications, and lower socioeconomic status and reduced access to prenatal care can exacerbate these disparities.^23^ As the largest payer for behavioral health services for IDD, covering more than 40% of births in the US, including 64% of deliveries to Black birthing people and 58% of deliveries to Hispanic birthing people,^24^ Medicaid plays a significant role in the potential to address the intersectional barriers birthing people with IDD encounter. The purpose of this study is to identify prenatal, childbirth, and postpartum outcomes in a diverse sample of birthing people with IDD enrolled in Medicaid in the US compared with people without IDD.

## Methods

### Data and Study Populations

For our retrospective cohort study of US pregnancies, data from the Centers for Medicare & Medicaid Services (CMS) for January 1, 2008, to December 31, 2019, including Medicaid Analytic eXtract and Transformed Medicaid Statistical Information System Analytic Files and personal summary and claims files, were used. The base study sample contained all Medicaid enrollees with IDD, defined as those with an autism spectrum disorder diagnosis (n = 1.7 million), intellectual disabilities (ID) diagnosis (n = 1.9 million), or autism spectrum disorder and ID diagnoses combined (n = 3.2 million). A person was categorized in the IDD group if they had at least 1 inpatient or 2 other claims associated with an autism spectrum disorder diagnosis (*International Classification of Diseases, Ninth Revision, Clinical Modification* [*ICD-9-CM*] codes 299.xx or *International Statistical Classification of Diseases and Related Health Problems, Tenth Revision, Clinical Modification* [*ICD-10-CM*] codes F84.x), or ID diagnosis (*ICD-9-CM* codes 317.xx-319.xx or *ICD-10-CM* codes F7x), or both, in alignment with previous IDD research.^25,26,27^ This study also included a randomly selected sample of 4.3 million Medicaid enrollees without IDD (autism spectrum disorder or ID; eFigure in Supplement 1). Algorithms published by Mathematica and CMS were used to identify pregnant and postpartum enrollees in the Medicaid data.^28,29^ As Medicaid claims for the study years do not capture gender identity, data were limited to birthing people with a gender denoted in Medicaid as female and do not include other birthing people who do not identify or who are not identified as female. The Drexel University institutional review board approved this study with a waiver for the use of claims data. This study followed the Strengthening the Reporting of Observational Studies in Epidemiology (STROBE) reporting guideline for reporting observational studies.

We identified claims related to live birth and organized them chronologically. We designated the date of the first recorded live birth claim as the conclusion of the initial episode. Subsequently, we instituted a washout period of 275 days to ascertain the end point for the subsequent episode. This approach facilitated the delineation of distinct episodes of pregnancy prior to a birth within our dataset. Next, we identified claims associated with miscarriage, stillbirth, or termination and labor and delivery, outcome unknown, organizing these by their respective dates. Claims outside a 275-day interval preceding or succeeding the live birth claims that were identified were preserved. Using the same criteria for selection, we determined the end points for these episodes. For the purposes of this study, only the first episode identified during the study period was analyzed. Medicaid eligibility type, by category, was defined based on the most common eligibility type during the observed enrollment time in the study years.

We purposively selected obstetric outcomes and medical and mental health conditions associated with and not associated with gestation with high and low prevalence that align with priorities of the population of individuals with IDD and with the goal of examining these conditions for the first time in a national US sample to inform future, comprehensive examinations (see the eTable in Supplement 2 for conditions and diagnosis codes). We adopted different temporal frames for obstetric outcomes and medical and mental health conditions used in previous research among the general population and people with IDD, including validated algorithms from the Chronic Condition Warehouse.^30,31,32^ Specifically, for the assessment of obstetric outcomes, we focused on the 275-day period immediately before and after the delivery end point. The analysis of co-occurring physical and mental health conditions extended to 1 year before and after the delivery end point. For survival analyses of postpartum anxiety and depression outcomes, the year preceding the delivery end point served as the washout period. Thus, for individuals without a claim for anxiety or depression in the year before delivery, the analysis commenced on the day after the delivery end point and continued until the 1-year anniversary of this end point.

### Statistical Analysis

Statistical analysis was performed from July 2023 to June 2024. Descriptive statistics of participant characteristics, including age at first observed delivery, were calculated by subgroup. Individuals with no race or ethnicity information were included in a separate “missing” category in both descriptive statistics and models. Race and ethnicity are captured within Medicaid eligibility data produced by CMS. Race variables were included in this study in comparison with disparities documented in previous research and in alignment with CMS-reported metrics of racial disparities in perinatal outcomes. The χ^2^ test and the *t* test were performed comparing IDD subgroups and the group without IDD. All obstetric and co-occurring physical and mental health conditions were compared across study groups in bivariate multivariable analyses. The latter comprised calculation of odds ratios for each condition comparing the group with IDD with the group without IDD using logistic regression, adjusting for age at delivery, year of delivery, race and ethnicity, and state of residence.

Time to postpartum anxiety and depression after delivery were examined using survival analysis. Individuals with no anxiety or depression claims in the year before delivery were followed up for 1 year after the delivery. Kaplan-Meier curves were constructed and hazard ratios calculated using Cox proportional hazards regression, adjusting for age at delivery, year of delivery, race and ethnicity, and state of residence. The proportional hazards assumption was visually tested using Kaplan-Meier curves and log-log survival plots. Analyses were conducted using SAS, version 15.2 (SAS Institute Inc) and R, version 4.2.0 (R Project for Statistical Computing). All *P* values were from 2-sided tests and results were deemed statistically significant at *P* < .05.

## Results

A total of 55 440 birthing people with IDD (8.2% of all Medicaid-enrolled people with IDD) and 438 557 females in the random sample of people without IDD, aged 14 to 50 years, had an observed delivery from 2008 to 2019. Among people with IDD, 714 (1.3%) were American Indian and Alaska Native, 885 (1.6%) were Asian or Other Pacific Islander, 17 805 (32.1%) were Black, 6603 (11.9%) were Hispanic or Latino, 26 361 (47.5%) were White, and 430 (0.8%) were multiracial; 17.9% were eligible for Medicaid based on poverty, and 61.8% were eligible based on disability (Table 1).

**Table 1.** Sample Characteristics

Characteristic	Participants, %			
	IDD			No IDD (n = 438 557)
	Total (n = 55 440)	Autism (n = 13 586)	ID, no autism (n = 41 854)	
Age at first delivery, mean (SD), y^a^^,^^b^	24.9 (6.7)	23.0 (5.9)	25.5 (6.8)	26.4 (6.3)
Age group (age at first delivery), y^a^^,^^b^				
14-20	33.0	46.2	28.7	21.6
21-34	58.2	48.7	61.3	67.5
35-50	8.7	5.2	9.9	10.9
Race and ethnicity^b^				
American Indian and Alaska Native	1.3	1.3	1.3	1.5
Asian or Other Pacific Islander	1.6	1.5	1.6	3.4
Black	32.1	24.7	34.5	22.8
Hispanic or Latino	11.9	10.1	12.5	29.1
Multiracial	0.8	0.6	0.8	0.4
White	47.5	57.2	44.4	39.4
Missing	4.8	4.6	4.8	3.4
Medicaid eligibility group^b^				
Poverty	17.9	23.3	16.2	56.1
Disability	61.8	42.1	68.2	5.9
Other	20.3	34.6	15.6	38.0

Abbreviations: ID, intellectual disabilities; IDD, intellectual and developmental disabilities.

^a^ *At first delivery* refers to the first delivery identified in these Medicaid claims data.

^b^ *P* < .05.

**Figure 1.**
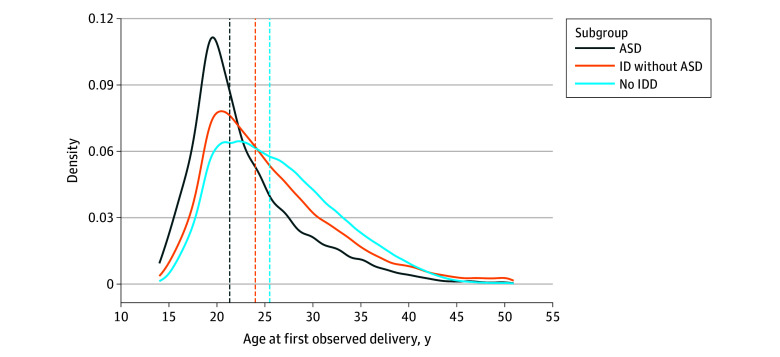
Age Distribution (Density Plot) and Median Age (Dashed Lines) for Birthing People at First Observed Delivery in Medicaid, 2008-2019 ASD indicates autism spectrum disorder; ID, intellectual disabilities; and IDD, intellectual and developmental disabilities.

Among all 55 440 birthing people with IDD with a delivery, the mean (SD) age at first delivery was 24.9 (6.7) years compared with 26.4 (6.3) years among all birthing people without IDD (Table 1). A total of 46.2% of autistic birthing people had a delivery before the age of 21 years compared with 28.7% of birthing people with ID and 21.6% of birthing people with no autism and no ID (Figure 1).

Among all types of births captured, the prevalence of live births was significantly lower among those with IDD (66.6%) (69.1% with autism; 65.8% with ID) compared with people without autism or ID (76.7%), with an adjusted odds ratio (AOR) for live birth of 0.83 (95% CI, 0.81-0.85) (Table 2). Accordingly, the prevalence of miscarriage, stillbirth, or termination was higher among individuals with IDD than people with neither autism nor ID (19.5% vs 15.3%).

**Table 2.** Birthing, Obstetric, and Co-Occurring Physical and Mental Health Conditions by Study Group

Outcome	All IDD (n = 55 440)		IDD subgroups				No IDD (n = 438 557), No. (%)
			Autism (n = 13 586)		ID, no autism (n = 41 854)		
	No. (%)	AOR (95% CI)	No. (%)	AOR (95% CI)	No. (%)	AOR (95% CI)	
Birthing outcomes							
Known live birth	36 931 (66.6)	0.83 (0.81-0.85)	9381 (69.1)	0.80 (0.76-0.83)	27 550 (65.8)	0.83 (0.81-0.86)	336 187 (76.7)
Known miscarriage, stillbirth, or termination	10 801 (19.5)	1.03 (1.00-1.06)	2586 (19.0)	1.09 (1.03-1.14)	8 215 (19.6)	1.00 (0.97-1.03)	67 012 (15.3)
Obstetric outcomes							
Gestational diabetes	5719 (10.3)	1.13 (1.09-1.17)	1379 (10.2)	1.14 (1.07-1.21)	4340 (10.4)	1.13 (1.08-1.18)	43 225 (9.9)
Gestational hypertension	4801 (8.7)	1.15 (1.10-1.20)	1209 (8.9)	1.12 (1.04-1.19)	3592 (8.6)	1.17 (1.11-1.22)	26 922 (6.1)
Preeclampsia	3368 (6.1)	1.17 (1.12-1.23)	767 (5.7)	1.10 (1.01-1.19)	2601 (6.2)	1.19 (1.13-1.26)	19 413 (4.4)
Co-occurring physical health conditions							
Allergies	6597 (11.9)	1.73 (1.66-1.79)	1523 (11.2)	1.87 (1.76-2.00)	5074 (12.1)	1.65 (1.58-1.72)	27 387 (6.2)
Asthma	9861 (17.8)	1.99 (1.84-1.97)	2428 (17.9)	1.95 (1.85-2.05)	7433 (17.8)	1.78 (1.72-1.85)	26 058 (5.9)
Epilepsy	5031 (9.1)	5.51 (5.17-5.88)	1002 (7.4)	4.13 (3.76-4.54)	4029 (9.6)	5.42 (5.05-5.82)	3376 (0.8)
Heart failure	800 (1.4)	1.84 (1.63-2.07)	145 (1.1)	1.81 (1.49-2.20)	655 (1.6)	1.62 (1.43-1.84)	1730 (0.4)
Hyperlipidemia	2920 (5.3)	2.17 (2.04-2.32)	530 (3.9)	2.07 (1.86-2.29)	2390 (5.7)	2.13 (1.99-2.28)	7288 (1.7)
Ischemic heart disease	814 (1.5)	1.96 (1.74-2.21)	151 (1.1)	2.02 (1.66-2.45)	663 (1.6)	1.75 (1.54-1.99)	1600 (0.4)
Kidney diseases	3142 (5.7)	1.49 (1.41-1.57)	690 (5.1)	1.28 (1.17-1.40)	2452 (5.9)	1.48 (1.39-1.57)	10 224 (2.3)
Obesity	9043 (16.3)	1.55 (1.50-1.60)	2005 (14.8)	1.48 (1.40-1.57)	7038 (16.8)	1.55 (1.49-1.61)	32 351 (7.4)
Co-occurring mental health conditions							
ADHD, conduct disorders, and hyperkinetic syndrome	8206 (14.8)	6.30 (6.00-6.61)	2708 (19.9)	7.10 (6.65-7.58)	5498 (13.1)	5.28 (4.99-5.58)	5498 (1.3)
Alcohol use disorders	2305 (4.2)	2.10 (1.96-2.25)	501 (3.7)	1.68 (1.51-1.87)	1804 (4.3)	2.02 (1.87-2.18)	5355 (1.2)
Anxiety disorders	15 475 (27.9)	2.94 (2.85-3.03)	4625 (34.0)	3.55 (3.39-3.71)	10 850 (25.9)	2.49 (2.40-2.58)	28 626 (6.5)
Bipolar disorder	13 507 (24.4)	4.55 (4.39-4.72)	3764 (27.7)	5.02 (4.77-5.29)	9743 (23.3)	3.79 (3.64-3.95)	12 774 (2.9)
Depressive disorders	17 775 (32.1)	3.26 (3.17-3.36)	4453 (32.8)	3.17 (3.03-3.31)	13 322 (31.8)	3.01 (2.91-3.10)	32 901 (7.5)
Drug use disorders	7164 (12.9)	1.52 (1.46-1.57)	1808 (13.3)	1.38 (1.30-1.46)	5356 (12.8)	1.42 (1.36-1.49)	23 581 (5.4)
Learning disabilities	2073 (3.7)	19.54 (16.98-22.47)	450 (3.3)	12.54 (10.26-15.33)	1623 (3.9)	20.35 (17.53-23.62)	331 (0.1)
Obsessive-compulsive disorder	644 (1.2)	4.83 (4.12-5.66)	334 (2.5)	9.47 (7.88-11.38)	310 (0.7)	2.38 (1.95-2.91)	584 (0.1)
Personality disorders	3213 (5.8)	6.35 (5.87-6.86)	954 (7.0)	5.58 (5.03-6.20)	2259 (5.4)	5.46 (4.99-5.98)	1868 (0.4)
Posttraumatic stress disorder	5287 (9.5)	4.08 (3.86-4.31)	1593 (11.7)	3.97 (3.69-4.29)	3694 (8.8)	3.38 (3.17-3.60)	5164 (1.2)
Schizophrenia	5046 (9.1)	8.90 (8.24-9.61)	988 (7.3)	5.30 (4.75-5.90)	4058 (9.7)	7.93 (7.30-8.62)	1679 (0.4)
Self-harm	435 (0.8)	4.59 (3.78-5.58)	108 (0.8)	4.25 (3.22-5.61)	327 (0.8)	4.12 (3.30-5.13)	455 (0.1)

Abbreviations: ADHD, attention-deficit/hyperactivity disorder; AOR, adjusted odds ratio; ID, intellectual disabilities; IDD, intellectual and developmental disabilities.

Compared with birthing people without IDD, birthing people with IDD had higher proportions of virtually all obstetric and co-occurring physical and mental health conditions, including gestational diabetes (10.3% vs 9.9%), gestational hypertension (8.7% vs 6.1%), preeclampsia (6.1% vs 4.4%), heart failure (1.4% vs 0.4%), hyperlipidemia (5.3% vs 1.7%), ischemic heart disease (1.5% vs 0.4%), obesity (16.3% vs 7.4%), anxiety disorders (27.9% vs 6.5%), depressive disorders (32.1% vs 7.5%), posttraumatic stress disorder (9.5% vs 1.2%), and asthma (17.8% vs 5.9%) (Table 2). These differences persisted after multivariable adjustment, and AORs for the obstetric conditions ranged between 1.13 (95% CI, 1.09-1.17) for gestational diabetes and 1.17 (95% CI, 1.12-1.23) for preeclampsia. Among the physical health conditions, co-occurring epilepsy among birthing people with IDD was associated with the largest AOR (5.51 [95% CI, 5.17-5.88]), while significant AORs for all the other physical conditions ranged between 2.17 (95% CI, 2.04-2.32) for hyperlipidemia to 1.49 (95% CI, 1.41-1.57) for kidney diseases. Co-occurring mental health conditions were associated with the highest AORs: schizophrenia, 8.90 (95% CI, 8.24-9.61); personality disorders, 6.35 (95% CI, 5.87-6.86) or attention-deficit/hyperactivity disorder or conduct disorders, 6.30 (95% CI, 6.00-6.61); bipolar disorder, 4.55 (95% CI, 4.39-4.72); PTSD, 4.08 (95% CI, 3.86-4.31); obsessive-compulsive disorder, 4.83 (95% CI, 4.12-5.66); anxiety disorders, 2.94 (95% CI, 2.85-3.03); depressive disorders, 3.26 (95% CI, 3.17-3.36); self-harm, 4.59 (95% CI, 3.78-5.58); alcohol use disorders, 2.10 (95% CI, 1.96-2.25); and drug use disorders, 1.52 (95% CI, 1.46-1.57).

**Figure 2.**
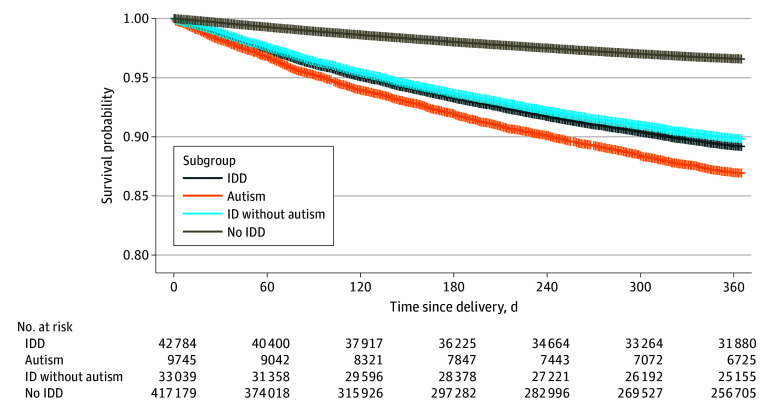
Kaplan-Meier and Cox Proportional Hazards Regression Results for Postpartum Anxiety ID indicates intellectual disabilities; IDD, intellectual and developmental disabilities.

**Figure 3.**
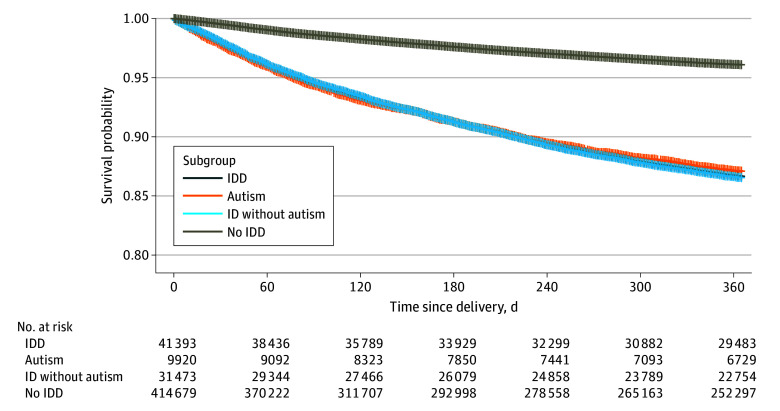
Kaplan-Meier and Cox Proportional Hazards Regression Results for Postpartum Depression ID indicates intellectual disabilities; IDD, intellectual and developmental disabilities.

The probabilities of postpartum anxiety or depression within a year of delivery among birthing people with IDD, overall and by subgroup, were increased compared with people without IDD (Figure 2 and Figure 3, respectively). In multivariable analysis, adjusted hazard ratios (AHRs) ranging from 2.6 (95% CI, 2.5-2.8) to 2.7 (95% CI, 2.5-2.7) were observed for both conditions among people with IDD. The probability of postpartum anxiety (AHR, 3.2 [95% CI, 2.9-3.4]) and postpartum depression (AHR, 2.4 [95% CI, 2.3-2.6]) was significantly higher among autistic people compared with people with ID only and people without IDD. (Figure 2).

## Discussion

Among more than 430 000 Medicaid-enrolled pregnancies and deliveries over a 12-year period among a diverse, nationwide sample of birthing people, we observed that birthing people with IDD were significantly younger at first delivery, had fewer live births, and had markedly higher rates of obstetric and co-occurring physical and mental health conditions in the year before and after delivery than their peers without IDD. The greatest disparities were observed in rates of co-occurring mental health conditions, yet all marked differences persisted after accounting for important covariates. Similar disparities were observed for the subgroups of autistic people and those with ID compared with people without IDD. The robust results clearly reveal a profile of a diverse group of birthing people with IDD who are both younger and substantially more burdened with serious obstetric and co-occurring health conditions than other birthing people in the US.

The younger age at delivery of the birthing people with IDD and the left shift of the age distribution of the subgroup of autistic people compared with people without IDD are important warning signs. There are reported gaps in the provision of reproductive health and planning, sexual education, and pregnancy-related knowledge to people with disabilities.^33^ Reproductive health education starting in adolescence for people and, when relevant, their caregivers and educating health care professionals, of the potential reproductive and co-occurring health concerns of this vulnerable population are underexamined topics. Centering people with IDD in future research is essential to understanding their experiences and needs, including prioritization of partnerships in developing reproductive education resources for people with IDD, support for their families in understanding and addressing their needs, and curricula for health care professionals.

Obstetric and co-occurring physical and mental health conditions were observed at high proportions among individuals with IDD before and after delivery. To prepare clinicians to treat and support this population, medical schools could incorporate education about these specialized needs into their curricula. Improved access to and adaptation of patient education to ensure that people with IDD are informed about medical and mental health risks and symptoms across the perinatal period is needed, in addition to referral options for obstetric care professionals for additional medical and mental health care. Community mapping across IDD and medical professionals could yield optimal localized connectedness. Research is needed to understand if practice and policy-level interventions, such as improving clinician knowledge of specialized needs of a patient population with IDD and broadening enrollment mechanisms to comprehensive health care, may improve these outcomes. The increased risk for postpartum anxiety and depression that we observed may be associated with an array of preexisting and newly occurring factors related to pregnancy, childbirth, and postpartum periods, including traumatic or adverse pregnancy experiences for people with IDD.^6,15^ Practice-level interventions could include administration of existing or adapted screening tools to identify the presence or potential risk for developing adverse conditions during the perinatal or postpartum period. There is evidence that integrating a validated depression screening tool, such as the Edinburgh Postnatal Depression Scale or the Postpartum Depression Screening Tool, throughout the perinatal period may help clinicians detect symptoms early and allow for referrals to mental health professionals.^34^ However, further research is needed to understand the utility of these screening tools for people with IDD.

Findings from this study highlight a need for attention on Medicaid in supporting birthing people with IDD throughout the perinatal period. More than 1 million people with IDD from diverse groups and communities rely on Medicaid for health insurance coverage, and supporting continued eligibility and enrollment throughout the postpartum period with links to medical, mental health, and behavioral health services is needed to improve outcomes and to reflect the combined service needs across individuals. Medicaid home- and community-based services (HCBS) waivers are a prominent source of enrollment options for people with IDD, as these programs include enrollment criteria that specifically include IDD and service options aligned to these individuals’ needs. Studies have found that these waivers can mitigate health disparities.^35^ However, coordination across HCBS waivers and medical care, including prenatal and postpartum care, is unclear, despite both being within the Medicaid system. As of February 2024, 44 states had extended Medicaid postpartum coverage from 60 days to 1 year post partum to ensure stable coverage during the critical postpartum period.^36^ Medicaid expansion efforts through the Patient Protection and Affordable Care Act have narrowed disparities among Black and Hispanic birthing people across outcomes, including maternal mortality, infant mortality, low birth weight, and preterm birth.^37^ When expansion is coupled with care coordination requirements for support needs of individuals with IDD, care experiences would likely be more fully linked. Our findings about the association of differences in postpartum care access and coordinated care with postpartum anxiety and depression is a key and urgent area for future research. Similarly, subsequent pregnancies and timing of additional pregnancies are important to understand among birthing people with IDD, relative to their perinatal outcomes and across groups.

Doula services have the potential to offer crucial support to people with IDD during pregnancy and childbirth, significantly reducing the risk of postpartum anxiety and depression, as observed in the general population.^38^ Medicaid coverage of doula services allows for cost savings by preventing the use of more costly crisis services that may occur when the health of people during and after pregnancy and that of their babies is not prioritized.^39^ This coverage offers benefits to vulnerable populations in low-income and disability groups served through Medicaid.

### Strengths and Limitations

The strengths of this study include a nationwide sample of Medicaid-enrolled birthing people with IDD and a representative comparison group of Medicaid enrollees without IDD over a 12-year period. The sample comprised the largest and most diverse group of birthing people with IDD to date and is the first US-specific examination of this group, to our knowledge, that aims to produce findings to inform future research, including adjustments for race and ethnicity and other characteristics.

Limitations of this study include those of claims-based research, such as the potential for selection bias related to inclusion and exclusion criteria and underreporting of birthing outcomes (eg, miscarriages and terminations). The inability to fully evaluate prenatal care and timing of presentation to pregnancy care, which can affect pregnancy outcomes, is limited in claims data and was outside the scope of the present study. Claims data include male or female sex, and the detection of nonbinary perinatal health care is limited, particularly during the study years. Care outside of the US Medicaid system was not observed, limiting generalizability to people with IDD with private health insurance. Contraception was not observed in the present study but may also affect pregnancy and postpartum outcomes, to the extent it can be examined using claims data.

## Conclusions

Findings from this cohort study present a need to tailor reproductive health education, perinatal care, and delivery services to ensure comprehensive and targeted support for birthing people with IDD. Designing and implementing policies aligned with and guided by the needs of people with IDD can lead to reductions in maternal health disparities. Advances to this objective may be accelerated by adapting current clinical guidelines and procedures associated with the specific needs and experiences of people with IDD. The effectiveness of new Medicaid policies in improving the health profile of birthing people with IDD, such as the postpartum coverage extension, should be assessed by tracking potential variation in outcomes after implementation of these policies.

## Notes

Supplement 1. **eFigure.** Sample Selection Flow Chart

Supplement 2. **eTable.** Conditions and Diagnosis Codes

Supplement 3.
Data Sharing Statement
