# Supplementary material for: Perinatal and Postpartum Health Among People With Intellectual and Developmental Disabilities
Source: JAMA Netw Open. 2024 Aug 15;7(8):e2428067. doi: 10.1001/jamanetworkopen.2024.28067 (PMC11327882; doi:10.1001/jamanetworkopen.2024.28067)
Supplement: Supplement 1. — eFigure. Sample Selection Flow Chart [file jamanetwopen-e2428067-s001.pdf]

Supplementary Online Content

Shea L, Sadowsky M, Tao S, et al. Perinatal and postpartum health among people with intellectual and developmental disabilities. *JAMA Netw Open.* 2024;7(8):e2428067. doi:10.1001/jamanetworkopen.2024.28067

eFigure. Sample Selection Flow Chart

This supplementary material has been provided by the authors to give readers additional information about their work.

© 2024 Shea L et al. *JAMA Network Open.*

eFigure. Sample Selection Flow Chart

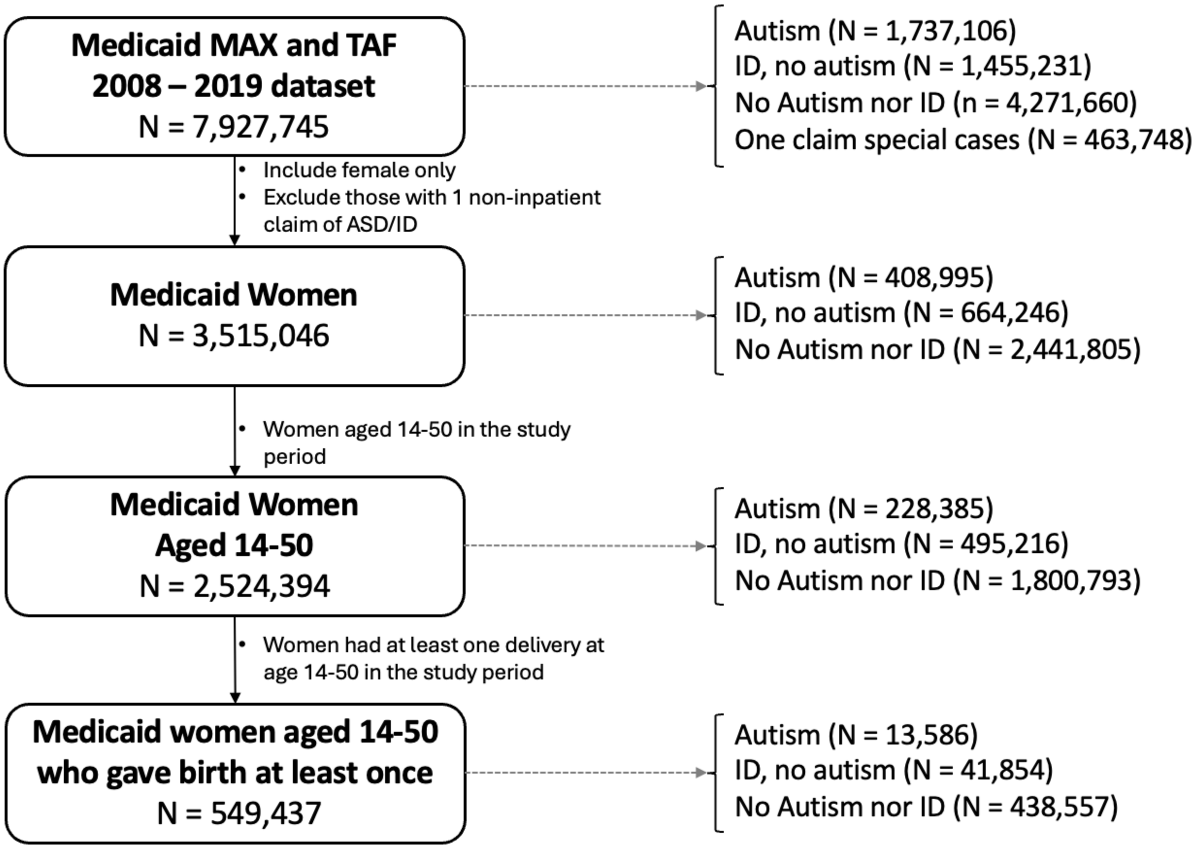

© 2024 Shea L et al. *JAMA Network Open.*
