# Supplementary material for: Perinatal and Postpartum Health Among People With Intellectual and Developmental Disabilities
Source: JAMA Netw Open. 2024 Aug 15;7(8):e2428067. doi: 10.1001/jamanetworkopen.2024.28067 (PMC11327882; doi:10.1001/jamanetworkopen.2024.28067)
Supplement: Supplement 3. — Data Sharing Statement [file jamanetwopen-e2428067-s003.pdf]

Data Sharing Statement

Shea. Perinatal and Postpartum Health Among People With Intellectual and Developmental Disabilities. *JAMA Netw Open*. Published August 15, 2024.
doi:10.1001/jamanetworkopen.2024.28067

Data
Data available: No

Additional Information
Explanation for why data not available: Access to claims data are governed by the Centers for Medicare and Medicaid Services (CMS) and the distribution of individual-level data is prohibited.
